# Adaptation to new nutritional environments: larval performance, foraging decisions, and adult oviposition choices in *Drosophila suzukii*

**DOI:** 10.1186/s12898-017-0131-2

**Published:** 2017-06-07

**Authors:** Nuno F. Silva-Soares, A. Nogueira-Alves, P. Beldade, Christen Kerry Mirth

**Affiliations:** 1 Instituto Gulbenkian de Ciência, Rua da Quinta Grande nº6, 2780-156 Oeiras, Portugal; 2 Université Toulouse III Paul Sabatier, Bâtiment 4R1, 118 Route de Narbonne, 31062 Toulouse Cedex 9, France; 3 School Biological Sciences, Monash University, 25 Rainforest Walk, Melbourne, VIC 3800 Australia

**Keywords:** *Drosophila suzukii*, *Drosophila biarmipes*, Foraging, Niche, Nutrition, Nutritional geometry

## Abstract

**Background:**

Understanding how species adapt to new niches is a central issue in evolutionary ecology. Nutrition is vital for the survival of all organisms and impacts species fitness and distribution. While most *Drosophila* species exploit rotting plant parts, some species have diversified to use ripe fruit, allowing earlier colonization. The decomposition of plant material is facilitated by yeast colonization and proliferation. These yeasts serve as the main protein source for *Drosophila* larvae. This dynamic rotting process entails changes in the nutritional composition of the food and other properties, and animals feeding on material at different stages of decay are expected to have behavioural and nutritional adaptations.

**Results:**

We compared larval performance, feeding behaviour and adult oviposition site choice between the ripe fruit colonizer and invasive pest *Drosophila suzukii*, and a closely-related rotting fruit colonizer, *Drosophila biarmipes*. Through the manipulation of protein:carbohydrate ratios in artificial diets, we found that *D. suzukii* larvae perform better at lower protein concentrations and consume less protein rich diets relative to *D. biarmipes*. For adult oviposition, these species differed in preference for substrate hardness, but not for the substrate nutritional composition.

**Conclusions:**

Our findings highlight that rather than being an exclusive specialist on ripe fruit, *D. suzukii*’s adaptation to use ripening fruit allow it to colonize a wider range of food substrates than *D. biarmipes*, which is limited to soft foods with higher protein concentrations. Our results underscore the importance of nutritional performance and feeding behaviours in the colonization of new food niches.

**Electronic supplementary material:**

The online version of this article (doi:10.1186/s12898-017-0131-2) contains supplementary material, which is available to authorized users.

## Background

The food substrates animals exploit play an important role in defining their ecological niche, determining their geographic distribution and abundance [1]. The possibility of exploiting new food substrates provides the opportunity for species to expand their distribution range and can contribute to diversification. To colonize new foods, animals need to adapt to the nutritional, physical, and chemical properties of the food [2]. Many studies have shown how this adaptation often involves acquiring the ability to exploit foods of differing physical properties or tolerate new, frequently toxic, chemical compositions [3, 4]. For example, the fruit fly *Drosophila sechellia* and tobacco hornworm *Manduca sexta* have evolved tolerance to toxic compounds of their food plants, *Morinda* and *Nicotiana*, respectively [3, 4]. Thus, the range of potential food types a species can exploit will define its ability to expand its nutritional niche [2]. In addition, to expand their nutritional niche species need to adapt to differences in the nutrient content of the new foods [2]. The nutritional composition of food substrates impacts a wide range of life-history and other traits [5–8]. Studies in grasshoppers [9] and caterpillars [10, 11] have shown that niche breadth greatly affects and is affected by an animal’s nutritional requirements and foraging strategies. Colonization of new nutritional substrates entails behavioural, physiological, and morphological adaptations that allow individuals to find, choose, and use that substrate. As such, a comprehensive understanding of how animals adapt to new foods requires combining analyses of how animals perform on different diets with analyses of the range of physical and non-nutritional properties animals can exploit [2]. Comparing the impact of diet composition on life history traits and food preferences between closely-related species with divergent nutritional niches can further our understanding of adaption to new nutritional resources and new habitats.

Species of the genus *Drosophila* offer a powerful system for an integrated analysis of the diversification of niche breath. *Drosophila* larvae explore a wide variety of food types and have a diverse range of foraging strategies [12]. The *melanogaster* species group, in particular, contains both generalist species, like *Drosophila melanogaster,* capable of colonizing several different kinds of rotting fruits and fungi, and specialized species, such as *D. sechellia*, that preferentially lays eggs in *Morinda* fruits, which are toxic to most other species [3]. *D. sechellia’s* colonization of this new niche has been accompanied by a number of physiological and behavioural adaptations, including changes in the olfactory system that resulted in aversive odors becoming attractive [13], increased tolerance to the toxins in *Morinda* [3], and reduced dopamine synthesis as the species relies heavily on food-derived dopamine [14]. Although *D. sechellia* is an elegant example of an adaption to a new food niche, it is difficult to disentangle adaptations to the toxins from adaptations to the new nutritional environment.

*Drosophila* species differ not only in the breadth of plant and fungal material, but also in the stage of substrate decay used for oviposition. Decaying organic matter like rotting fruits are dynamic environments that change in nutrient composition (notably, protein and carbohydrate content), pH, and microbial communities throughout the rotting process [15, 16]. Yeasts, crucial to the rotting process and the main source of protein for Drosophilid flies [17–19], colonize decaying organic matter in a species-specific sequential manner [16–18] and increase in concentration as fruits rot. Because differences in oviposition preference dictate the environment in which larvae will develop [20], species-specific preferences in the order of colonization provide an opportunity to explore how species partition nutritional resources.

The Southeast-Asian *D. suzukii* (Matsumura) is unusual among *Drosophila* in its preference for colonizing ripe, rather than rotting, soft-skinned fruits such as strawberries [21]. In recent years, *D. suzukii* has become an invasive pest species, both in Europe and North America, causing severe agricultural damage to several fruit growing industries [21–23]. Studies of *D. suzukii*’s adaptation to ripe fruit have focused on the adult females’ large and serrated ovipositor that can pierce and lay eggs under the ripe fruit’s skin [24]. Despite its economical relevance and potential threat to the environment, little is known about how *D. suzukii* larvae cope with the low protein nutritional environment of ripe fruit. The closely-related *Drosophila biarmipes* (Malloch), unable to pierce ripe fruit skin and limited to colonizing rotting fruit [24], provides a good reference for comparison to *D. suzukii* to explore how adaptation to a new temporal niche reflects nutritional requirements, larval feeding behaviour, and oviposition strategies.

Here, we make use of *D. suzukii* and *D. biarmipes* to understand how differences in their nutritional niche affect the response of life history traits to the macronutrient composition of the larval diet, and how that correlates with larval feeding behaviour and adult oviposition preferences. We use Nutritional Geometry [5, 25, 26] to test the hypothesis that *D. suzukii* larvae perform better in diets with lower protein content when compared to *D. biarmipes*. This approach uses artificial diets varying the quantities of two nutrients to determine how various life history traits map to the nutrient space, and has been applied to animals across many taxa [7–9, 11, 27–31]. Then, we use behavioural assays to test how larvae choose their feeding substrates and how adults choose oviposition sites. We find that differences in the response of larval life history traits and feeding choices reflect divergence in the macronutrient requirements between species, whereas oviposition choice reflects differences in the range of substrates a species can exploit.

## Methods

### Fly stocks

We used the L19 strain of *D. suzukii* generously provided by Vincent Debat (French Natural History Museum, Paris), and the 14023-0361.11 strain of *D. biarmipes* obtained from the *Drosophila* Species Stock Center (San Diego). *D. suzukii* were maintained at 22.5 °C, and *D. biarmipes* at 25 °C both on standard laboratory fly food: 45 g of molasses, 75 g of sucrose, 70 g of cornmeal, 10 g of agar, 1100 ml of water, and 25 ml of a 10% Nipagin solution per litre. For the maintenance of *D. suzukii*, we added strips of autoclaved paper as perching sites, and dry yeast to increase egg production.

### Protein and sugar quantification in strawberries

To quantify the protein and sugar content in decaying strawberries, we placed single ripe strawberries into 11 plastic cups and inserted these cups into fly population cages (11 × 20.5 × 27 cm) at 25 °C. We replicated this three times. To simulate natural yeast inoculation, we introduced 50 males (25 *D. suzukii* and 25 *D. biarmipes)* inside each cage where a 6 cm diameter Petri dish containing standard fly food and live-yeast paste (Baker’s yeast) provided a source of yeast during the first 2 days of the experiment. From each replicate, we froze (−20 °C) one strawberry per day on days 1–10 and on day 13 after the start of the yeast inoculation. Samples were later thawed and lysed using glass beads in a Qiagen TissueLyser (10 min at maximum speed). After centrifugation (10 min at 6000×*g*), we measured protein (Pierce BCA Protein assay kit; Thermo Scientific #23227) and glucose/sucrose (Glucose and Sucrose colourimetric/fluorimetric assay kit; Sigma #MAK013) concentrations in the supernatant, following each manufacturer’s instructions but using half-size reactions.

To characterize changes in macronutrient content of strawberries as they decayed, we log (x+1) transformed the P and C concentration data and used a linear mixed effect model, with replicate as the random effect and day as fixed effect as in [30].

### Larval performance assays

Using nutritional geometry, we raised larvae of *D. biarmipes* and *D. suzukii* on 24 different diets varying in protein, carbohydrate, and caloric content. For each diet, we mixed solutions of yeast (Lesaffre SAF-Instant Red) and sucrose (Sidul, Santa Iria de Azóia, Portugal) that were one of four concentrations (45, 90, 180, and 360 mg/ml) with 0.5% agar corresponding to generate diets of four different caloric concentrations (0.18, 0.36, 0.72 and 1.44 kcal/ml, respectively). For each caloric concentration, we mixed the solutions to produce six different protein to carbohydrate (P:C) ratios: 1.5:1 (corresponding to pure yeast solution), 1:1, 1:2, 1:4, 1:8, and 1:16. All foods were autoclaved and we added 1% of propionic acid (Acros organics, Geel, Belgium) and 1% solution of nipagin (10% p-hydroxy benzoic acid methyl ester in 95% ethanol) to prevent fungal and bacterial contamination. This experiment was conducted at 25 °C and replicated four times for each study species. We measured five life-history traits: survival from first instar to pupariation, larval development time (checked twice per day, at 10:00 and 18:00), female and male pharate adult weight, and ovariole number. Pharate adult weight and ovariole number were measured as described in [8].

### Larval foraging assays

We performed two types of larval foraging assays: a two-choice assay where larvae were offered the choice between two P:C ratios, and a no-choice assay where larvae fed on a single diet. Prior to the assays, larvae were reared on standard fly food in densities of approximately 200 individuals in 6 cm Petri dishes filled with standard fly food. We collected L3 (third larval instar) larvae 0–5 h post ecdysis for all assays.

For the two-choice assays, we used 9.2 cm split Petri dishes with each half filled with one food type. We placed ten L3 larvae on each side of the plate (total of 20 per plate) and allowed them to forage for either 2 or 4 h at 25 °C in the dark. We offered larvae one of three choice pairs of foods equal in caloric content (0.72 kcal/ml) but differing in P:C ratios: 1.5:1 vs 1:8; 1:1 vs 1:8; 1:1 vs 1:16. Each choice pair and time point was replicated 10 times. To distinguish between the diets offered, we dyed foods with either red or blue food dye (1% Rayner, Billingshurst, UK), switching the colours between replicate assays to control for colour preference. At the end of the assay, we collected larvae and scored gut colour content of individual larvae followed by the spectrophotometric analysis of the larval pooled gut contents of each replicate. All larvae from each replicate were transferred into one 2 ml Eppendorf and frozen at −20 °C. We later extracted the dye from the sample by adding 80 µl of ice-cold methanol, homogenizing the sample with Qiagen TissueLyser (1 min at maximum speed), and centrifuging at 13,000×*g* for 10 min at 4 °C. We measured the absorbance of 60 µl of the supernatant from each sample at 450 nm for the red dye and at 600 nm for the blue using a Victor3 multilabel plate reader (Perkin Elmer, Waltham, USA).

The no-choice assay design was similar, except that larvae were left to forage on a single P:C ratio (either 1.5:1, 1:1, 1:2, 1:4, 1:8 or 1:16). All foods were isocaloric (0.72 kcal/ml) and dyed blue for the subsequent quantification of the amount of food ingested by spectrophotometric analysis.

### Adult oviposition assays

We performed two types of oviposition preference assays: choice between food P:C ratios and choice between food hardness. From each species, we selected 20 newly-eclosed females and 10 newly-eclosed males from density controlled bottles. To transfer *D. suzukii* flies between vials, we are required by regulation to anesthetise them with CO_2_. To minimize the effects of exposure to CO_2_ on oviposition behavior for both species, we sorted twenty virgin females and ten males into vials and allowed them to mate for 7–9 days. This resulted in the death of some of the females, mainly due to the animals getting stuck on the food. As a result, we had a variable number of females in each vial for the oviposition assay. To adjust for this variability, we report the number of eggs laid normalized by number of females in the vial. Further, we used only vials that contained a minimum of 10 surviving females and 5 surviving males to run 25 replicates of each preference assay. Flies from individual vials were transferred to assay arenas containing three 15 ml Falcon tube lids with food, glued onto a Petri dish (6 cm) capped by a 200 ml plastic cup. All foods were isocaloric (0.72 kcal/ml), but were either of one of three P:C ratios (1:1, 1:4, and 1:8) or of different agar concentrations (1, 2, and 3%). Assays were run at 25 °C for 6 h in complete darkness. At the end of the assay, we froze all flies and assessed oviposition preference by counting the number of eggs in each food patch and dividing it by the number of females in the assay arena.

### Statistical analyses

All statistical analyses were done in R (version 3.0.2, R Development Core Team 2013, https://www.r-project.org/). All datasets and scripts are publically available from Figshare (doi: 10.4225/03/58ca18ae80d1a).

From the nutritional geometry experiment, we estimated the response of each life history trait to the protein, carbohydrate, and caloric content of the food based on the methods described in [6, 8, 30]. For survival, we fit a generalized linear model with a quasi-binomial distribution, to account for the overdispersion of the data, and with a logit link function. For the remaining traits, we fit linear mixed effect models, with replicate as the random effect. The effect of both the linear and quadratic components of carbohydrate and protein, as well as their cross-product, was included in our models. To visualize each trait’s response on the macronutrient space defined by our panel of diets, we used non-parametric thin plate splines. To compare response shapes between traits and between species, we standardized the dependent variable values to a mean of zero and a unit standard deviation, and compared responses using partial F tests.

The two-choice larval assays provided two types of data: number of larvae with blue and/or red colour in their guts (scored by eye), and quantification of each colour in pooled larvae (determined by spectrophotometer absorbance). For the dataset of larval gut colour (scored by eye) we calculated the proportion of larvae in each replicate that ate protein-rich food only, protein-poor food only, both foods, and no food. We then tested for differences for proportion of individuals in these categories between: (1) choice pairs; (2) species; (3) time points; and (4) possible interactions; by fitting a generalized linear model with a quasi-poisson distribution to account for overdispersion of the data.

For the spectrophotometer absorbance data, we calculated the proportions of protein-rich versus protein-poor diet consumed based on the proportion of measured absorbance values for each dye. We then tested for differences in the proportion of protein-rich food using: choice pairs; species; time points; and possible interactions as factors; by fitting a generalized linear model with a quasi-poisson distribution. For the proportion of protein-rich food in the gut for each treatment and each species, we then tested for significant departure from the null hypothesis (no preferences for either protein rich or protein poor foods: µ = 0.5) using Wilcoxon signed rank test. We also compared the amounts of protein-rich food ingested between species for each treatment by pair-wise comparison of least squared means. We adjusted for multiple comparisons using sequential Bonferroni (Holm) correction of the p values (α = 0.05).

The no-choice larval assays allow us to use the quantity of food larvae ingest in each diet to assess how they regulate macronutrient intake [5]. To do this, we calculated quantity of ingested protein and carbohydrate in each diet, by measuring the absorbance values for the blue dye extracted from the gut. By comparing protein and carbohydrate intake across diets, we can determine how larvae prioritise their macronutrient intake. To linearize the data, we applied the log(x+1) transformation. To compare macronutrient intake between treatments and between species, we first normalized protein and carbohydrate intake by species by subtracting the species-specific median from each sample [26]. Thus, a value of zero indicates there is no nutritional offset relative to the median, while positive and negative offsets represent excess or deficits in the intake, respectively (as in [32]). We then analysed the effect of species, P:C ratio, and assay time on each macronutrient offset using linear models. We chose to pool the data of both time points, even if time had a significant effect for protein consumption, because the main macronutrient differences between species were relative to carbohydrates (Additional file 4: Tables S1, Additional file 5: Table S2). We compared the different macronutrient offsets slopes by comparison of the least squared trends, adjusting for multiple comparisons with sequential Bonferroni (Holm) correction.

Finally, we assessed adult oviposition preference in relation to substrate macronutrient composition and substrate hardness in two separate assays. For each assay, to test for differences in the proportion of eggs laid (1) between each food type, (2) between species, and (3) respective possible interactions, we used generalized linear model, with a quasi-binomial distribution to account for overdispersion. We compared the proportion of eggs laid in each substrate against a null distribution of 33% (no preference between the three substrates offered) by comparing least squared means, adjusting for multiple comparisons using sequential Bonferroni (Holm) correction.

## Results

### Larval nutritional performance matches each species use of fruit decay stage

We started by measuring protein and sugar content of strawberries, one of the preferred oviposition substrates of *D. suzukii* [33], over the course of 14 days to verify our assumption that P:C ratio is lower in ripe fruits and increases with the rotting process (Additional file 1: Figure S1). Similarly to what had been shown for figs [30], we found that protein concentrations significantly increased with rotting (linear mixed effect model, p value = 0.045, R^2^ = 0.191), while sugar concentrations significantly decreased (p value <0.001, R^2^ = 0.533). This corresponded to significant increases in P:C ratio with decay (p value <0.001, R^2^ = 0.460).

*D. suzukii* prefers to lay its eggs on ripe fruits while *D. biarmipes* colonizes fruits at later stages of decay. Given the changes in P:C during fruit decay, we hypothesized that, relative to *D. biarmipes, D. suzukii* would perform better at lower P:C ratios and/or have higher tolerance to high concentrations of carbohydrates/low concentrations of protein. To test this, we quantified the response of five larval life history traits (survival, developmental time, male/female body mass, and ovariole number) across 24 diets varying in protein, carbohydrate, and caloric composition. We equated better performance with the highest values for survival, body mass and ovariole number, and lowest values for development time. We analysed the effects of protein and carbohydrate content on each trait, and compared response surfaces between traits and species.

For both species, few individuals reached the pupal stage on 1:16 P:C foods (regardless of the caloric value), with survival below 2% for *D. suzukii* and below 7% for *D. biarmipes*. Also for both species, we found that the highest mean survival was obtained on 1.5:1 P:C foods but at different caloric contents: 0.36 kcal/ml for *D. suzukii* (77% survival) and 1.44 kcal/ml for *D. biarmipes* (96% survival). A summary of the trait value ranges can be found in Tables S3 and S4 (Additional files 6, 7). On average, *D. biarmipes* larvae developed faster (105 h), had more ovarioles (26.3 ovarioles), and were larger (1.13 mg male and 1.42 mg females) when raised on the highest protein concentrations (Additional file 7: Table S4). For *D. suzukii*, larvae developed fastest (131 h), had the most ovarioles (24.5), and grew into largest flies (1.47 mg male and 1.82 mg female) when raised in intermediate protein concentrations (Additional file 6: Table S3). When testing the effect of macronutrient composition on the different traits, we found that protein content, both linear and quadratic components, contributed significantly to all life history traits in both species (Table 1). On the other hand, carbohydrate content only played a significant role for *D. biarmipes* male body mass (linear and quadratic components) and female body mass (in interaction with protein content).

**Table 1:** The linear and quadratic effects of carbohydrate (C) and protein (P), and their cross product, in the larval diet on five life history traits: survival, developmental time, female and male adult mass and ovariole number in *Drosophila suzukii* and *Drosophila biarmipes*

Trait		C	p	C^2^	p^2^	C × p	R^2^
*D. suzukii*							
Survival							
β		−0.002	*0.107*	<−0.001	<−*0.001*	<−0.001	–
t value		−0.315	11.287***	−1.401	−10.979***	−0.26	
Dev. time							
β		0.095	−*2.122*	<−0.001	*0.009*	0.001	0.54
t value		0.816	−11.77***	−0.341	9.833***	1.074	
Female mass							
β		0.003	*0.019*	<−0.001	<−*0.001*	<−0.001	0.36
t value		1.983	8.58***	−1.365	−7.377***	−1.355	
Male mass							
β		0.001	*0.013*	<−0.001	<−*0.001*	<−0.001	0.41
t value		1.284	8.19***	−1.262	−7.288***	−0.457	
Ovariole no.							
β		0.016	*0.107*	<−0.001	<−*0.001*	<−0.001	0.09
t value		1.172	5.017***	−0.718	−3.84***	−1.61	
*D. biarmipes*							
Survival							
β		−0.004	*0.126*	<−0.001	<−*0.001*	<−0.001	–
t value		−1.051	13.236***	−1.24	−12.438***	−0.074	
Dev. time							
β		0.184	−*3.617*	<0.001	*0.016*	<−0.001	0.74
t value		1.26	−14.58***	1.669	12.09***	−0.581	
Female mass							
β		0.001	*0.117*	<−0.001	<−*0.001*	<−*0.001*	0.66
t value		1.9	13.273***	−1.756	−9.049***	−2.512*	
Male mass							
β		*0.001*	*0.007*	<−*0.001*	<−*0.001*	<−0.001	0.54
t value		2.491*	8.972***	−2.801**	−5.977***	−0.707	
Ovariole no.							
β		−0.002	*0.186*	<−0.001	<−*0.001*	< 0.001	0.43
t value		−0.188	10.849***	−1.52	−9.486***	0.498	

For all traits with exception of survival, the models were linear mixed-effects models fit by maximum likelihood. Survival data was analysed with a generalized linear model, assuming a quasi-binomial distribution of survival probabilities and a logit link. Significant coefficients are in italics

* p < 0.05, ** p < 0.01, *** p < 0.001

**Fig. 1:**
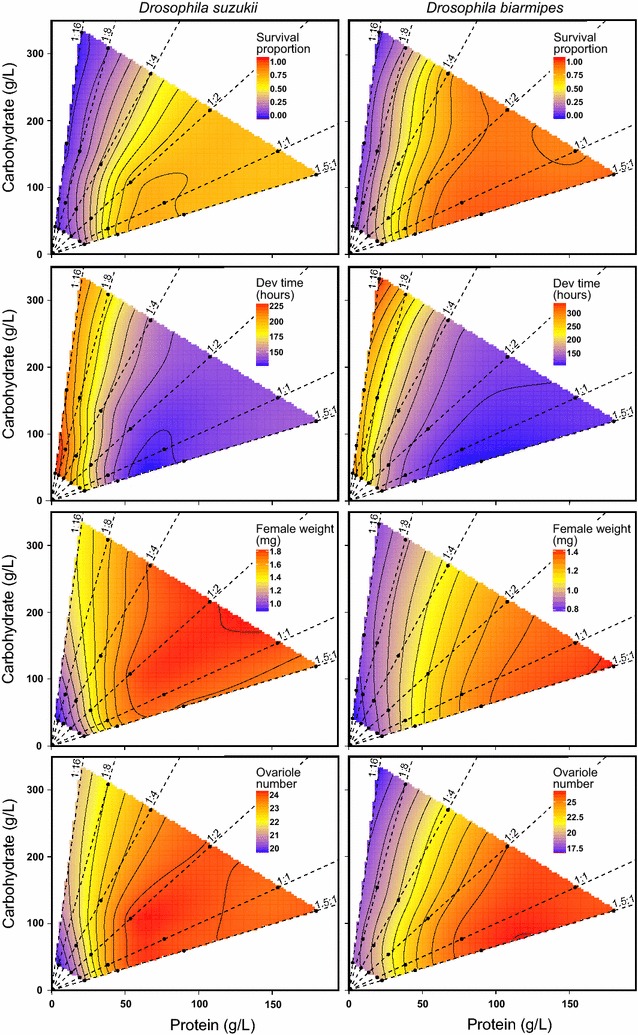
The effects of protein and carbohydrate content of the larval diet on four larval life-history traits of *D. suzukii* (*left column*) and *D. biarmipes* (*right column*). The fitted response surfaces of the effects of 24 different diets varying in protein, carbohydrate, and caloric composition for: (*first row*) proportion of larvae surviving from first instar larvae to pupae; (*second row*) developmental time from first instar larvae to pupae; (*third row*) female pharate weight; and (*fourth row*) total number of ovarioles of adult females. *Dashed black lines* represent the P:C ratios. We replicated four times each block of 24 diets per species using 30 first instar larvae per diet. *Filled black circles* represent the respective nutritional coordinates of each of the 24 diets used (if a dot is absent not enough larvae survived that treatment to measure the trait)

Figure 1 represents the response of the different life history traits to our macronutrient landscape (male mass in Additional file 2: Figure S2). For *D. suzukii*, we found similar response surfaces for all traits (Additional file 8: Table S5), with performance generally increasing between low and intermediate protein levels and plateauing at intermediate protein levels and high P:C ratios. For *D. biarmipes*, all traits were maximized at high protein level and high P:C ratios, but differed in the shape of the response to macronutrient variation. We found three groups of significantly different responses in *D. biarmipes* traits (Additional file 9: Table S6). In Fig. 1 this is particularly clear in relation to how the traits responded to increasing protein levels: survival increased steadily until reaching a plateau, developmental time decreased first steeply and later more shallowly until its minimum, and body mass (both male and female) increased without ever reaching a plateau within our macronutrient panel. The response for ovariole number was statistically indistinguishable from that of both survival and developmental time. When we compared trait responses between species, we found differences for all traits, with the exception of survival (Table 2).

**Table 2:** Differences in the response surfaces between *D. suzukii* and *D. biarmipes* for each trait

Trait	D. freedom	L ratio	*p* value
Survival	5	1.793	0.877
Dev. time	5	*58.999*	*<0.001*
Female weight	5	*72.124*	*<0.001*
Male weight	5	*21.968*	*<0.001*
Ovariole no.	5	*32.213*	*<0.001*

Using partial F tests, we compared the response surfaces generated from linear mixed effects models on the scaled parameter values. Response surfaces that show significant differences are highlighted in italics

### When compared to *D. suzukii, D. biarmipes* larvae prefer and consume more protein

We ran two types of behavioural assays to test the hypotheses that macronutrient regulation differs between *D. suzukii* and *D. biarmipes* larvae (no-choice assay) and that larvae choose and consume foods that maximize their performance (two-choice assay).

**Fig. 2:**
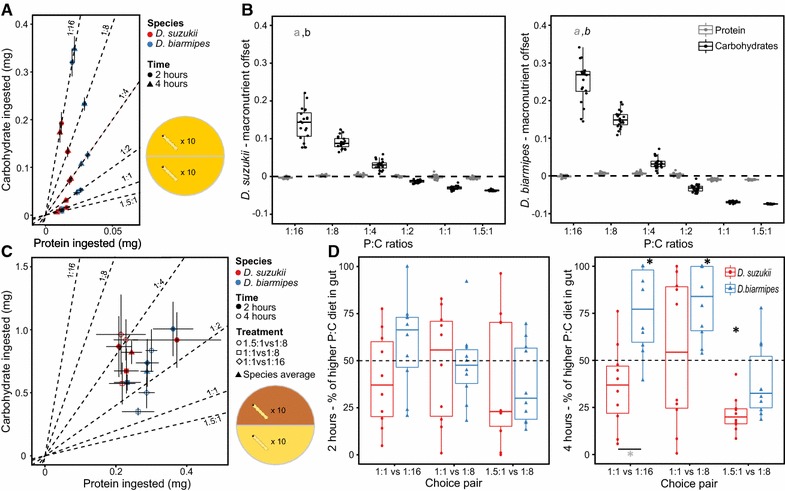
*D. suzukii* and *D. biarmipes* differ in the quantity of food eaten depending on the diet. **A** Amount of protein and carbohydrate ingested in the no-choice assay. Twenty larvae were offered one of six P:C ratios and were able to forage for 2 or 4 h.* Each* *dot* represents the mean value of 10 replicates and the *error bars* are 95% confidence intervals of the means. **B** Differences between protein and carbohydrates offsets in *D. biarmipes* and *D. suzukii*. Each condition was replicated ten times (both time points were pooled). *Dashed line* represents the normalized median for each macronutrient (0 = no macronutrient offset). Differences in font type (*regular* versus* italic*) between letters represent significant differences across least squared trends for *a* protein and *b* carbohydrates between the two species. **C** Amount of protein and carbohydrate ingested in the two-choice assay.* Each* *dot* represents the mean value of 10 replicates, except the *triangles*, which represent the average intake target for both species calculated from the pooled data of all treatments/time points. *Error bars* are 95% confidence intervals of the means. Twenty larvae were offered a choice between two protein to carbohydrate (P:C) ratios in three different combinations. Larvae were able to forage for 2 or 4 h and each time point was replicated ten times. The quantity of food in the larval gut was determined by spectrophotometer. **D** The figures show the percentage of the total amount of food ingested that corresponded to the higher protein food (1:1 for the first and second food pairs, and 1.5:1 for the third food pair) found in the guts of *D. suzukii* and *D. biarmipes* larvae in the two-choice assay. *Black asterisks* represent significant differences to no choice (50%-*dashed black line*—see Additional file 13: Table S10) and* grey asterisks* represent significant differences between species for the same diet (Least squared means comparison, see Additional file 14: Table S11)

In our no-choice experiment (Fig. 2A, B), where larvae were left to feed on only one of six P:C ratios (1.5:1; 1:1; 1:2; 1:4; 1:8; and 1:16), we observed that *D. biarmipes* had higher median protein (0.022 mg) and carbohydrate (0.082 mg) intake than *D. suzukii* (0.015 mg protein, 0.044 mg carbohydrate). We then compared the two species in terms of the intake offset for each macronutrient from its normalized median of 0 (Fig. 2B, for mean consumption values see Additional file 10: Table S7). Briefly, an offset value of 0 represents no deviation from the species-specific median across all foods for the respective macronutrient. Conversely, positive or negative offset values represent higher or lower consumption, respectively, of that macronutrient relative to the median (see “Methods”). Low nutrient offsets and respective variation across treatments indicate a strong nutrient intake regulation and the same logic applies to the reverse situation. Our analysis revealed that both *D. suzukii* and *D. biarmipes* regulate protein consumption over carbohydrate consumption; protein intake deviates significantly less from the median than carbohydrate intake (Fig. 2A), absolute consumption values in Fig. 2A). We also observed that deviations in protein and carbohydrate consumption differed between the two species: *D. biarmipes* had steeper slope for the offset of carbohydrates consumed than *D. suzukii* (Fig. 2A), suggesting a stronger response to P:C variation and higher food consumption rate (Fig. 2B).

We then assessed whether *D. suzukii* and *D. biarmipes* larvae chose differently when given a choice between a protein rich and a protein poor diet of equal caloric value (1.5:1 vs 1:8; 1:1 vs 1:8; and 1:1 vs 1:16—Fig. 2C, D). Our data on the colour inside the gut of individual larvae revealed that, for both species, larvae tended to not mix foods and fed preferentially on the protein rich food, with a higher proportion of larvae doing so in *D. biarmipes* relative to *D. suzukii* (Additional file 3: Figure S3; Additional file 11: Table S8). This result agrees with our no-choice assay results where larvae prioritized protein over carbohydrate regulation. Our spectrophotometric dataset, allowing the quantification of protein and carbohydrate ingested in pooled larva, showed significant differences between the two species after 4 h of choice assay (Fig. 2D, and absolute consumption values in Fig. 2C). With the exception of when given a choice between 1.5:1 and 1:8 foods, *D. biarmipes* larvae always consumed significantly more of the protein-rich food (Fig. 2D, see Additional file 12: Table S9, Additional file 13: Table S10). On the other hand, *D. suzukii* larvae consumed as much protein-rich as protein-poor food (Fig. 2D), showing no preference between available P:C ratios. The exception was when given a choice between 1.5:1 and 1:8 foods: in this case *D. suzukii* larvae avoided 1.5:1 (pure yeast) food.

### Adult oviposition preference correlates with substrate hardness rather than substrate nutritional status

**Fig. 3:**
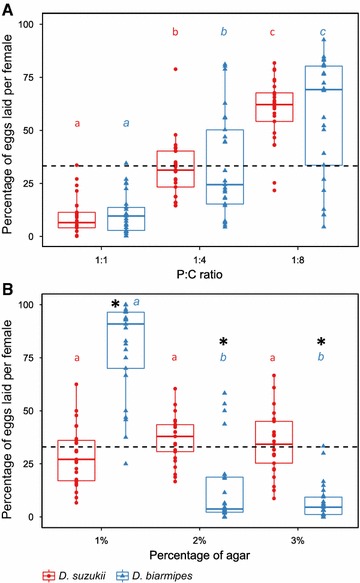
*D. suzukii* and *D. biarmipes* have the same oviposition preference for P:C ratios, but differ for substrate hardness. The oviposition site preference was estimated by the percentage of eggs laid in each diet. The* letters* (*red* for *D. suzukii*,* blue italics* for *D. biarmipes*) indicate significant differences in the proportion of eggs laid between different diets within a species, with significant differences marked by* different letters*, as determined by least squared means assuming no-choice value of 33% (*dashed line* in* all panels*—see Additional file 14: Table S11, Additional file 15: S12). Each treatment was replicated 25 times. *Stars* represent significant differences from the null hypothesis of 33%. **A** Females were offered a choice between three diets differing in their P:C ratios for oviposition. **B** Females were offered a choice between three diets differing in their hardness (agar concentration) for oviposition

The nutritional response surfaces for life history traits (Fig. 1) and nutritional preference of L3 larvae (Fig. 2D) suggested that *D. suzukii* both perform better on lower protein concentrations and ingest less protein than *D. biarmipes*. We next hypothesized that adult females would choose to lay their eggs in food substrates more suitable for their larvae. To test this hypothesis, we assessed oviposition site preference by letting flies choose between three isocaloric P:C ratios for oviposition: 1:1, 1:4, and 1:8. Our prediction was that *D. biarmipes* would prefer to lay eggs in the protein-rich food (1:1) with P:C ratios closer to those of rotting fruit, while *D. suzukii* would prefer oviposition substrates with lower P:C ratios, closer to those of ripe fruit. However, we found that *D. biarmipes* and *D. suzukii* did not differ in preference. They both preferred the lowest P:C ratio (1:8) for oviposition, and laid the fewest eggs on the highest P:C ratio (Fig. 3A; Table 3; Additional file 14: Table S11).

**Table 3:** *D. suzukii* and *D. biarmipes* show significant preferences in oviposition for P:C ratios and react differently to hardness

	D. freedom	Pr(chi)
Nutritional preference		
P:C ratios	2	<0.001***
Species	1	1.000
Food*species	2	0.828
Hardness preference		
Agar %	2	<0.001***
Species	1	1.000
Food*species	2	<0.001***

We fit the data with generalized linear models using a quasi-binomial distribution to account for the overdispersion of the data

Significant differences are highlighted in italics: * p < 0.05, ** p < 0.01, *** p < 0.001

We next turned our attention to another property that differs between ripe and rotting fruit. *D. suzukii* females have piercing ovipositors that are unique among *Drosophila* flies and allow them to penetrate the harder skin of ripe fruits [24]. This lead us to hypothesize that substrate hardness might underlie differences in oviposition site preferences between the two species. To test if *D. biarmipes* and *D. suzukii* make oviposition decisions based on substrate hardness, we gave females the choice between 1:8 P:C ratio foods differing in agar concentration: 1, 2, and 3%. Our prediction was that *D. suzukii* would prefer higher agar concentrations relative to *D. biarmipes.* We found that, while *D. suzukii* females did not show any preference between food patches, laying equal proportions of eggs in each of the three, *D. biarmipes* showed a clear preference for the softest food (1% agar) (Fig. 3B; Table 3; Additional file 15: Table S12).

## Discussion

In this study, we aimed to understand how new nutritional niches impact nutritional performance and foraging behaviour. We studied these in two closely related species of *Drosophila* that differ in the order in which they colonize fruit; *D. suzukii* that colonizes ripe fruit and *D. biarmipes* that colonizes rotting fruit. Given this difference and the changes in protein and carbohydrate composition as fruit decays (Additional file 1: Figure S1), we hypothesized that *D. suzukii* larvae would have better performance in and prefer, lower P:C ratios when compared to *D. biarmipes* larvae.

### Effects of dietary macronutrient composition on larval life-history traits

Supporting our hypothesis, we found that *D. suzukii* maximizes performance for all life history traits examined at lower protein concentrations when compared to *D. biarmipes*. *D. suzukii* responded similarly across all traits, surviving better, developing faster and into larger flies with more ovarioles at intermediate protein concentrations. This is in agreement with a previous study showing absence of trade-offs for *D. suzukii* life history traits for larvae on protein-poor diets [34]. In contrast, *D. biarmipes* larvae survived better, and developed faster and into larger flies with more ovarioles at the highest protein concentrations. Similar differences in the response of life history traits to diet composition have previously been observed between *Drosophila simulans* and *Zaprionus indianus,* two species that co-inhabit fig plantations in southern Brazil but colonize figs at different stages of decay [30]. This presents the interesting possibility that the order of fruit colonization correlates with larval nutritional performance across *Drosophila* species, with early colonizers performing better at lower P:C ratios when compared to late colonizers.

Fruits, either ripe or rotten, are complex substrates with several macro and micronutrients varying across fruit types/species and decaying stage. In our study, we changed protein concentrations by manipulating yeast content of our artificial diets. However, as in rotting fruits, yeast contributes more than protein to the diet. Because of this, we cannot disentangle the effect of changes in protein from changes in other nutrients provided by the yeast. For future studies, it will prove valuable to use a synthetic medium, such as developed by [35], to distinguish effects of changes in protein and carbohydrates from changes in other nutrients. Also, analyses of the effect of dietary P:C ratio variation on other life-history traits, such as egg production rate and lifetime fecundity, might uncover relevant trade-offs for the ecology of these fruit flies. Lastly, even though nutritional geometry is a powerful analytical tool, it cannot fully recreate the changes in dietary conditions of rotting fruit. It is, thus, possible that in our artificial setting we either diminish or enhance differences in performance between species. Further studies exploring effects of other properties of rotting fruit, such as changes in concentration of chemical by-products of the rotting process and in microbial composition, as well as interspecific competition would certainly provide a more detailed characterization of the differences in how *D. suzukii* and *D. biarmipes* use food resources.

### Larval nutritional preferences and macronutrient compromise

We expected the differences in the response of life history traits to nutrition to be reflected in the larval foraging preferences. While larvae have limited mobility and usually are restricted to forage on the fruit where females laid their eggs, rotting fruits are not homogeneous and larvae might be able to choose between patches with different nutritional composition [16, 20]. Indeed, we found that, when given the choice between two foods with different P:C ratios, the rotting fruit colonizer *D. biarmipes* consumed more of the protein rich diets (in two of three choices provided), while ripe fruit colonizer *D. suzukii* consumed indistinguishable amounts of protein-rich and protein-poor foods (Fig. 2D). In contrast, when we scored individual larval preferences (Additional file 3: Figure S3), we found that, for both species, more larvae chose protein rich food than protein poor food, and rarely mixed the diets. Taken together, these datasets imply that these species use different foraging strategies when offered a choice between food types. While *D. biarmipes* larvae select between food types, in *D. suzukii* larvae the preference for protein rich food was weaker and rather then choosing the protein rich food they compensated by altering their food intake when feeding on protein poor diets. The latter observation contrasts with results from *D. melanogaster*, whose larvae mix between unbalanced foods to achieve an intermediate P:C ratio [8]. This suggests interesting differences in foraging strategies between these three species, with *D. suzukii* and *D. biarmipes* preferring to forage in a single food and regulating their macronutrient intake through the volume of food ingested.

We also found that both *D. suzukii* and *D. biarmipes* were more affected by the protein than by the carbohydrate concentrations (Table 1). This was reflected in their foraging strategies on single foods: both species prioritized protein intake at the expense of consuming excess carbohydrates (Fig. 2A). However, compared to *D. biarmipes* larvae, *D. suzukii* consumed less protein (Fig. 2A, B). When examining normalized macronutrient intakes (Fig. 2A), we found that the offset for carbohydrate intake showed steeper responses to changes in diet P:C ratio in *D. biarmipes* when compared to *D. suzukii* (Fig. 2A). This suggests that rather than simply differing in ingestion rates, *D. suzukii* and *D. biarmipes* differ in macronutrient intake target [36].

### Adult oviposition preferences and niche exploration

For *Drosophila* females, yeast represents an important cue for oviposition substrate selection [37]. This selection is crucial as it defines the environment for the next generation of larvae. We expected *D. suzukii* and *D. biarmipes* females to make oviposition choices that reflected the differences in larval nutritional performance and macronutrient preferences. However, we found that these species did not differ in their preference for P:C ratios for oviposition (Fig. 3A). This is in contrast to what had been described for another fly species pair that differ in order of fruit colonization, *Zaprionus indianus* (earlier colonizer of figs) and *D. simulans* (later colonizer) [30]. *Z. indianus* preferred to oviposit on low P:C ratios and *D. simulans* preferred higher P:C ratios but only when competing with *Z. indianus.* This suggests that *D. suzukii* and *D. biarmipes* use different cues to distinguish their order of colonization than do *Z. indianus* and *D. simulans*.

Both *D. suzukii* and *D. biarmipes* preferentially laid their eggs on substrates with low P:C ratios (1:8), similar to what had been previously described for *D. melanogaster* [8, 20]. These ratios are suboptimal for larval performance for all three species [8, 20]. However, because fruits continue to change as they ripen and then rot, the P:C ratio larvae will be eating is likely to be higher from that chosen for oviposition [8, 20]. It seems likely that both *D. biarmipes* and *D. suzukii* are following a similar strategy as *D. melanogaster*: choosing oviposition sites that will be beneficial to larvae with time.

Despite the absence of ovipositional differences between *D. suzukii* and *D. biarmipes* relative to the nutritional composition of the substrate (Fig. 3A), they still differed in oviposition preference relative to another relevant physical property of decaying fruits, substrate hardness (Fig. 3B). *D. biarmipes* preferred laying their eggs in the softest medium while *D. suzukii* females showed no preference for any hardness (Fig. 3B). Thus, it seems that the unique ovipositor of *D. suzukii* widens the range of potential substrates it can exploit allowing this species to use resources unavailable to other species of *Drosophila* [24]. This is also consistent with the natural spatial distribution for *D. suzukii* female flies that exploit oviposition sites on tree crowns, contrasting with other species that only use fruit that has fallen on the ground [38]. Furthermore, this illustrates two alternative strategies for interspecific competition during oviposition connected to different dimensions of the nutritional niche [2]; based on macronutrient content between *Z. indianus* and *D. simulans* [30] and determined by substrate hardness in *D. suzukii* and *D. biarmipes* (this study).

## Conclusions

Thanks to its modified ovipositor, *D. suzukii* has the unique capability of colonizing ripe fruit, inaccessible to other *Drosophila* species like *D. biarmipes*, which are restricted to rotting fruit. Using nutritional geometry, we reveal differences between these species going beyond the ovipositor and extending to differences in the nutritional responses of life history traits, larval foraging behaviour, and adult oviposition preference. Our findings highlight that rather than being an exclusive specialist on ripe fruit, *D. suzukii* has a generalist profile relative to food exploitation, being able to colonize a wider range of food substrates than *D. biarmipes*, which is limited to higher P:C ratios and soft substrates (i.e. rotting fruits). Altogether, these results match well with the predictions proposed by Machovsky-Capusta et al. [2] for a successful invasive species [2]. *D. suzukii* not only has a flexible performance relative to the dimensions of macronutrient performance and food composition, but also excels at food exploitation among *Drosophila* flies. For the future, it would be interesting to extend this analysis not only to other *Drosophila* species, but also to other taxa. The *Drosophila* clade provides significant molecular resources with *D. melanogaster* and has other species with interesting ecological contexts, such as the specialist *D. sechellia*, allowing us to address both evolutionary and mechanistic questions relative to nutritional adaptation. By analyzing other taxa including other invasive species, our capacity to predict/control the damage of biological invasions will improve. These studies ultimately would provide more profound insight into phenotypic adaptation to new foods.

## Additional files

**Additional file 1: Figure S1.** The macronutrient composition of strawberries changes with the stage of decay. The plots show the log transformations of protein (top left) and sugar (sucrose and glucose) amounts in ug per μl (top right) and protein to sugar ratio (bottom left) over the course of 14 days in rotting strawberries for three replicates. Black lines indicate the regression estimates from linear models and the grey shaded areas represent 95% confidence intervals.

**Additional file 2: Figure S2.** The effects of protein and carbohydrate content of the larval diet on male adult mass of *D. suzukii* (left) and *D. biarmipes* (right). The fitted response surfaces of the effects of 24 different diets varying in protein, carbohydrate, and caloric composition for male pharate weight as proxy for male adult mass. Dashed black lines represent the P:C ratios. Filled black circles represent the respective nutritional coordinates of each of the 24 diets used (if a dot is absent, not enough larvae survived that treatment to measure the trait).

**Additional file 3: Figure S3.** *D. suzukii and D. biarmipes* choose protein rich food over a period of 2 and 4 hours. Twenty larvae were offered a choice between two protein to carbohydrate (P:C) ratios in three different combinations (1:1/1:16, 1:1/1:8 and 1.5:1/1:8). Twenty larvae were left to forage for two or four hours and each treatment was replicated ten times per time point. Larval food preference was first assessed by eye to determine the colour of the larval gut. Each larva was assigned to one of four possible categories: protein rich food; carbohydrate rich food; both foods and none. (Top row) The percentage of *D*. *suzukii* and *D*. *biarmipes* larvae assigned to each category after two hours of foraging (Bottom row).

**Additional file 4: Table S1.** Effects of diet, time, larval species and possible interactions in the amount of protein that third instar larvae ingested for the no-choice larval assays.

**Additional file 5: Table S2.** Effects of diet, time, larval species and possible interactions in the amount of carbohydrates that third instar larvae ingested for the no-choice larval assays.

**Additional file 6: Table S3.** Mean values and standard deviation (StDev) for each trait for each diet of the nutritional geometry for *D. suzukii.*

**Additional file 7: Table S4.** Mean values and standard deviation (StDev) for each trait for each diet of the nutritional geometry for *D. biarmipes.*

**Additional file 8: Table S5.** Pairwise comparisons between the response surfaces of the five life history traits in *Drosophila suzukii.*

**Additional file 9: Table S6.** Pairwise comparisons between the response surfaces of the five life history traits in *Drosophila biarmipes.*

**Additional file 10: Table S7.** Mean values (mg) and standard deviation (StDev) for protein and carbohydrate intake for each P:C ratio of the no-choice experiment.

**Additional file 11: Table S8.** Effects of pair of diets presented, time, larval species and possible interactions for the amount of larvae that chose: Protein rich food; Carbohydrate rich food; Both foods; and None. We analyzed our data with generalized linear models using a quasi-possion distribution (ANOVA type II).

**Additional file 12: Table S9.** Effects of pair of diets presented, time, larval species and possible interactions in the amount of protein rich food that third instar larvae chose to consume. We analyzed our data with a generalized linear model using a quasi-poisson distribution (ANOVA type II).

**Additional file 13. Table S10.** Differences of consumption of each diet for each treatment in *D. suzukii* and *D. biarmipes*. We used Wilcoxon signed ranked test each to compare the percentage of protein-rich diet present in the gut to 50% (no-choice). Treatments that show significant differences are highlighted in bold.

**Additional file 14: Table S11.** Least squared means (Lsmean), standard errors (St. error), and groups for *D. suzukii* and *D. biarmipes* nutritional oviposition assays, with significant differences denoted by different numbers in the group column (adjusting p-values using the Bonferroni method for a significance level of 0.05).

**Additional file 15. Table S12.** Least squared means (Lsmean), standard errors (St. error), and groups for *D. suzukii* and *D. biarmipes* hardness oviposition assays, with significant differences denoted by different numbers in the group column (adjusting p-values using the Bonferroni method for a significance level of 0.05).
