# Supplementary material for: Adaptation to new nutritional environments: larval performance, foraging decisions, and adult oviposition choices in Drosophila suzukii
Source: BMC Ecol. 2017 Jun 7;17:21. doi: 10.1186/s12898-017-0131-2 (PMC5463304; doi:10.1186/s12898-017-0131-2)
Supplement: Supplementary file 1 — Additional file 1: Figure S1. The macronutrient composition of strawberries changes with the stage of decay. The plots show the log transformations of protein (top left) and sugar (sucrose and glucose) amounts in ug per μl (top right) and protein to sugar ratio (bottom left) over the course of 14 days in rotting strawberries for three replicates. Black lines indicate the regression estimates from linear models and the grey shaded areas represent 95% confidence intervals. [file 12898_2017_131_MOESM1_ESM.docx]

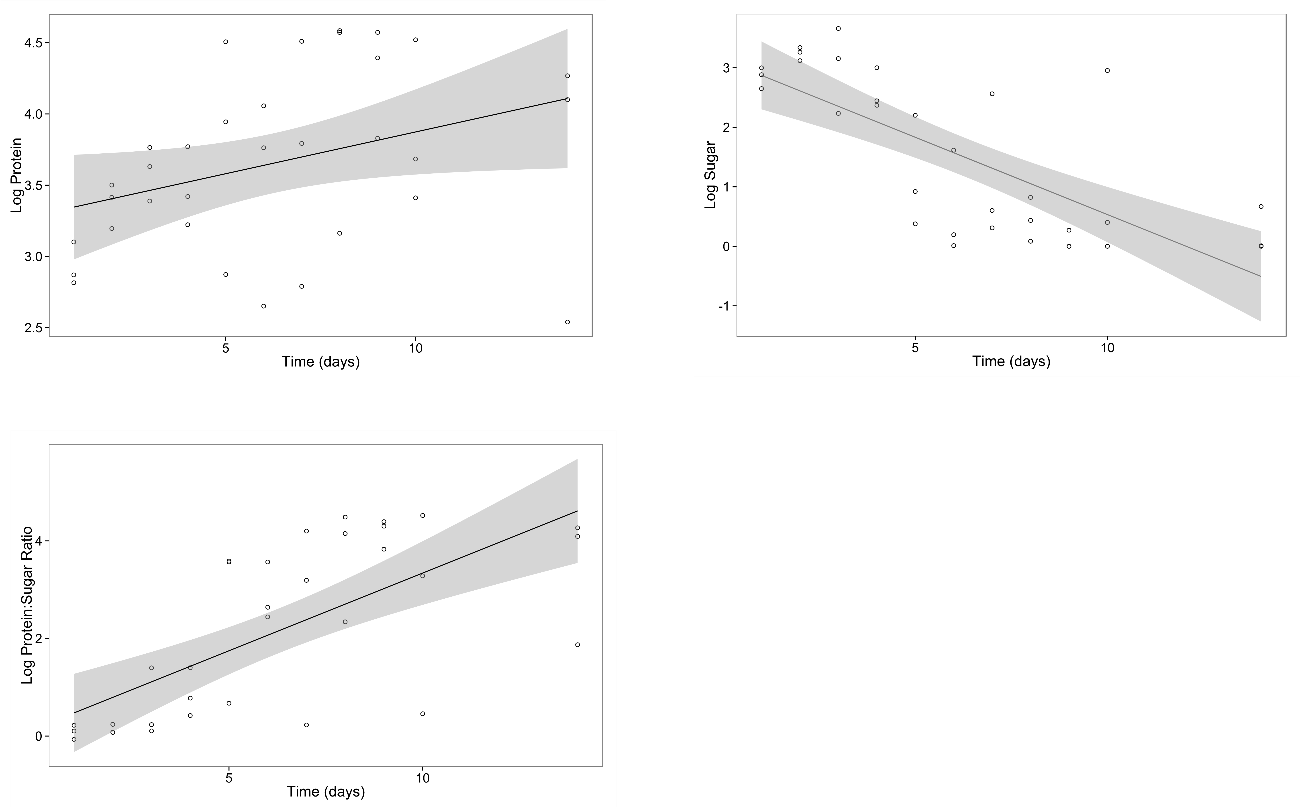


Figure S1 – The macronutrient composition of strawberries changes with the stage of decay. The plots show the log transformations of protein (top left) and sugar (sucrose and glucose) amounts in ug per μl (top right) and protein to sugar ratio (bottom left) over the course of 14 days in rotting strawberries for three replicates. Black lines indicate the regression estimates from linear models and the grey shaded areas represent 95% confidence intervals.
