# Supplementary material for: Adaptation to new nutritional environments: larval performance, foraging decisions, and adult oviposition choices in Drosophila suzukii
Source: BMC Ecol. 2017 Jun 7;17:21. doi: 10.1186/s12898-017-0131-2 (PMC5463304; doi:10.1186/s12898-017-0131-2)
Supplement: Supplementary file 2 — Additional file 2: Figure S2. The effects of protein and carbohydrate content of the larval diet on male adult mass of D. suzukii (left) and D. biarmipes (right). The fitted response surfaces of the effects of 24 different diets varying in protein, carbohydrate, and caloric composition for male pharate weight as proxy for male adult mass. Dashed black lines represent the P:C ratios. Filled black circles represent the respective nutritional coordinates of each of the 24 diets used (if a dot is absent, not enough larvae survived that treatment to measure the trait). [file 12898_2017_131_MOESM2_ESM.docx]

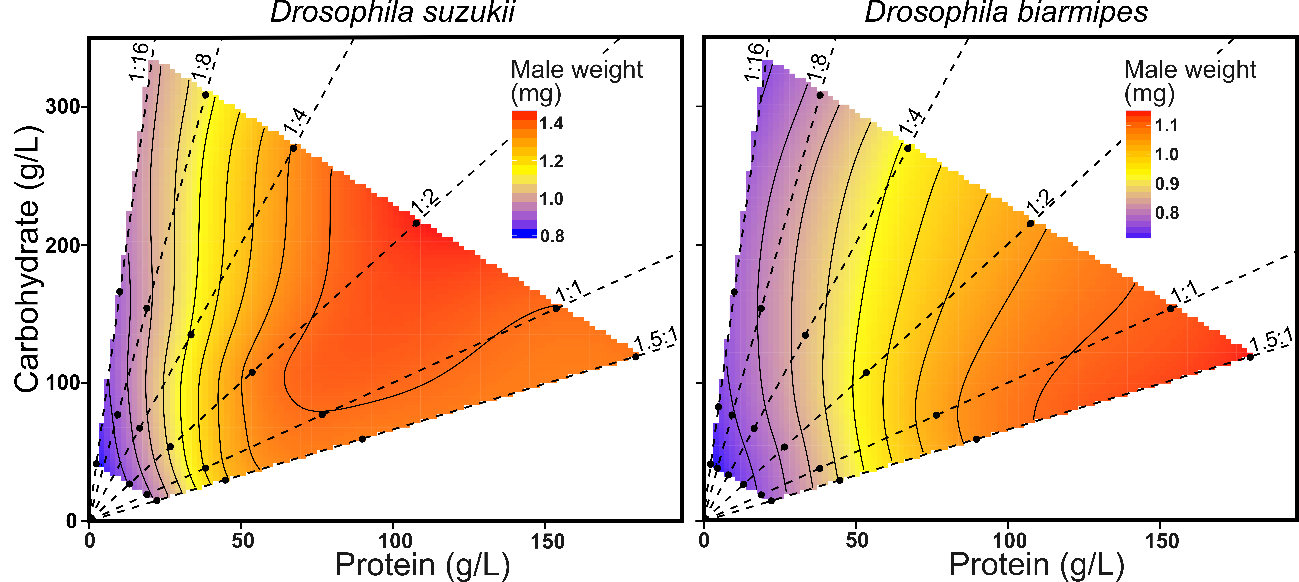


Figure S2 - The effects of protein and carbohydrate content of the larval diet on male adult mass of *D. suzukii* (left) and *D. biarmipes* (right). The fitted response surfaces of the effects of 24 different diets varying in protein, carbohydrate, and caloric composition for male pharate weight as proxy for male adult mass. Dashed black lines represent the P:C ratios. Filled black circles represent the respective nutritional coordinates of each of the 24 diets used (if a dot is absent not enough larvae survived that treatment to measure the trait).
