# Supplementary material for: Adaptation to new nutritional environments: larval performance, foraging decisions, and adult oviposition choices in Drosophila suzukii
Source: BMC Ecol. 2017 Jun 7;17:21. doi: 10.1186/s12898-017-0131-2 (PMC5463304; doi:10.1186/s12898-017-0131-2)
Supplement: Supplementary file 3 — Additional file 3: Figure S3. D. suzukii and D. biarmipes choose protein rich food over a period of 2 and 4 hours. Twenty larvae were offered a choice between two protein to carbohydrate (P:C) ratios in three different combinations (1:1/1:16, 1:1/1:8 and 1.5:1/1:8). Twenty larvae were left to forage for two or four hours and each treatment was replicated ten times per time point. Larval food preference was first assessed by eye to determine the colour of the larval gut. Each larva was assigned to one of four possible categories: protein rich food; carbohydrate rich food; both foods and none. (Top row) The percentage of D. suzukii and D. biarmipes larvae assigned to each category after two hours of foraging (Bottom row). [file 12898_2017_131_MOESM3_ESM.docx]

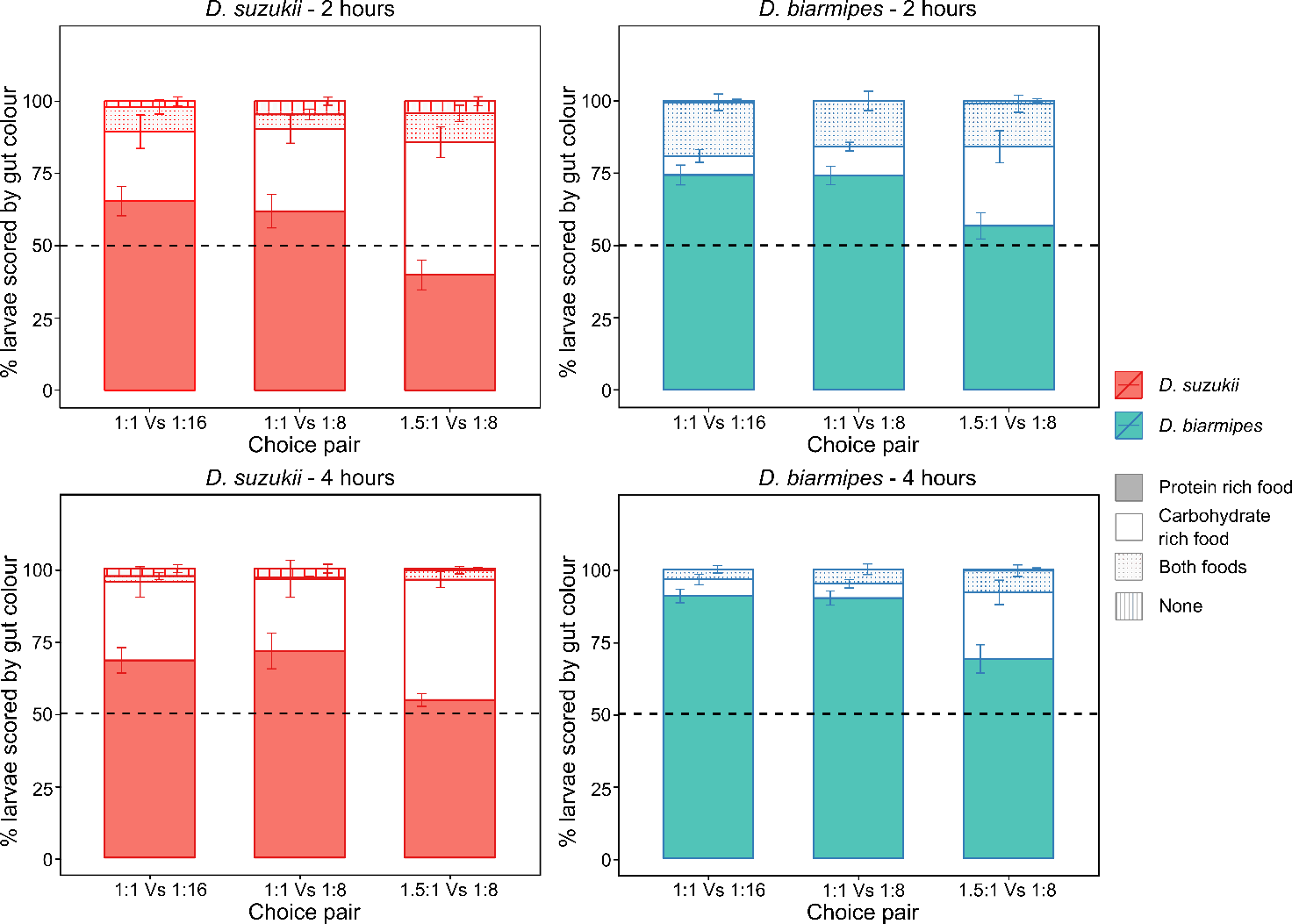


Figure S3 - D. suzukii and D. biarmipes choose protein rich food over a period of 2 and 4 hours. Twenty larvae were offered a choice between two protein to carbohydrate (P:C) ratios in three different combinations (1:1/1:16, 1:1/1:8 and 1.5:1/1:8). Twenty larvae were left to forage for two or four hours and each treatment was replicated ten times per time point. Larval food preference was first assessed by eye to determine the colour of the larval gut. Each larva was assigned to one of four possible categories: protein rich food; carbohydrate rich food; both foods and none. (Top row) The percentage of D. suzukii and D. biarmipes larvae assigned to each category after two hours of foraging (Bottom row).
