# Supplementary material for: Adaptation to new nutritional environments: larval performance, foraging decisions, and adult oviposition choices in Drosophila suzukii
Source: BMC Ecol. 2017 Jun 7;17:21. doi: 10.1186/s12898-017-0131-2 (PMC5463304; doi:10.1186/s12898-017-0131-2)
Supplement: Supplementary file 5 — Additional file 5: Table S2. Effects of diet, time, larval species and possible interactions in the amount of carbohydrates that third instar larvae ingested for the no-choice larval assays. [file 12898_2017_131_MOESM5_ESM.docx]

**Table S2** –Effects of diet, time, larval species and possible interactions in the amount of carbohydrates that third instar larvae ingested for the no-choice larval assays.

|  | **Df** | **Pr(>F)** |
| --- | --- | --- |
| Time | 1 | 0.84 |
| Diet | 1 | **< 0.001 ***** |
| Species | 1 | 0.095 |
| Time*Diet | 1 | 0.916 |
| Time*Species | 1 | 0.807 |
| Diet*Species | 1 | **< 0.001 ***** |
| Time*Diet*Species | 1 | 0.816 |

We analyzed our data with a linear model (ANOVA type II: F_7,232_=55.36, adjusted R^2^=0.61). Level of significance: *p* < 0.05 * ; *p* < 0.01 ** ; *p* < 0.01 ***
