# Supplementary material for: Adaptation to new nutritional environments: larval performance, foraging decisions, and adult oviposition choices in Drosophila suzukii
Source: BMC Ecol. 2017 Jun 7;17:21. doi: 10.1186/s12898-017-0131-2 (PMC5463304; doi:10.1186/s12898-017-0131-2)
Supplement: Supplementary file 6 — Additional file 6: Table S3. Mean values and standard deviation (StDev) for each trait for each diet of the nutritional geometry for D. suzukii. [file 12898_2017_131_MOESM6_ESM.docx]

**Table S3** - Mean values and standard deviation (StDev) for each trait for each diet of the nutritional geometry for *D. suzukii.*

| **Food** | | **Survival** | | **Dev time** | | **Male mass** | | **Female mass** | | **Ovariole** | |
| --- | --- | --- | --- | --- | --- | --- | --- | --- | --- | --- | --- |
| **P:C** | **Cal.** | **Mean** | **StDev** | **Mean** | **StDev** | **Mean** | **StDev** | **Mean** | **StDev** | **Mean** | **StDev** |
| 1:16 | 45 | 0.02 | 0.02 | 264.00 | 101.82 | 1.08 | NaN | - | - | - | - |
| 1:8 | 45 | 0.01 | 0.02 | 216.00 | NaN | - | - | 1.04 | NaN | 19.00 | NaN |
| 1:4 | 45 | 0.01 | 0.02 | 264.00 | NaN | - | - | 0.87 | NaN | - | - |
| 1:2 | 45 | 0.04 | 0.03 | 188.80 | 23.05 | 1.07 | NaN | 1.17 | NaN | 16.00 | NaN |
| 1:1 | 45 | 0.14 | 0.10 | 184.47 | 45.38 | 0.86 | 0.29 | 1.03 | 0.23 | 18.67 | 4.16 |
| 1.5:1 | 45 | 0.16 | 0.06 | 192.00 | 27.71 | 0.62 | 0.18 | 0.89 | 0.33 | 20.33 | 2.52 |
| 1:16 | 90 | 0.00 | 0.00 | - | - | - | - | - | - | - | - |
| 1:8 | 90 | 0.07 | 0.07 | 240.00 | 30.54 | 0.68 | 0.13 | 0.73 | 0.26 | - | - |
| 1:4 | 90 | 0.11 | 0.11 | 202.46 | 33.53 | 0.90 | 0.24 | 0.99 | 0.19 | 18.50 | 0.71 |
| 1:2 | 90 | 0.31 | 0.08 | 164.32 | 23.29 | 1.06 | 0.23 | 1.38 | 0.23 | 22.00 | 2.00 |
| 1:1 | 90 | 0.63 | 0.11 | 149.65 | 19.54 | 1.23 | 0.21 | 1.61 | 0.26 | 23.64 | 2.48 |
| 1.5:1 | 90 | 0.77 | 0.05 | 136.00 | 10.54 | 1.31 | 0.13 | 1.63 | 0.28 | 24.06 | 1.46 |
| 1:16 | 180 | 0.02 | 0.03 | 232.00 | 11.31 | 0.82 | NaN | - | - | - | - |
| 1:8 | 180 | 0.01 | 0.02 | 216.00 | NaN | 0.71 | NaN | - | - | - | - |
| 1:4 | 180 | 0.34 | 0.07 | 161.95 | 22.11 | 1.11 | 0.18 | 1.48 | 0.38 | 22.36 | 2.34 |
| 1:2 | 180 | 0.76 | 0.08 | 138.55 | 11.43 | 1.40 | 0.13 | 1.79 | 0.22 | 24.50 | 1.99 |
| 1:1 | 180 | 0.71 | 0.13 | 131.01 | 15.22 | 1.39 | 0.17 | 1.78 | 0.27 | 23.83 | 1.74 |
| 1.5:1 | 180 | 0.69 | 0.09 | 134.36 | 11.70 | 1.36 | 0.18 | 1.70 | 0.22 | 23.95 | 1.79 |
| 1:16 | 360 | 0.00 | 0.00 | - | - | - | - | - | - | - | - |
| 1:8 | 360 | 0.03 | 0.03 | 192.00 | 19.60 | 0.95 | 0.01 | 1.41 | 0.18 | 22.00 | NaN |
| 1:4 | 360 | 0.43 | 0.07 | 149.02 | 6.78 | 1.37 | 0.21 | 1.77 | 0.24 | 23.30 | 1.89 |
| 1:2 | 360 | 0.70 | 0.17 | 139.33 | 10.98 | 1.47 | 0.09 | 1.81 | 0.23 | 24.05 | 1.84 |
| 1:1 | 360 | 0.69 | 0.10 | 139.08 | 13.21 | 1.39 | 0.17 | 1.82 | 0.19 | 23.78 | 1.90 |
| 1.5:1 | 360 | 0.72 | 0.07 | 140.93 | 10.71 | 1.38 | 0.17 | 1.67 | 0.28 | 24.00 | 1.41 |
