# Supplementary material for: Adaptation to new nutritional environments: larval performance, foraging decisions, and adult oviposition choices in Drosophila suzukii
Source: BMC Ecol. 2017 Jun 7;17:21. doi: 10.1186/s12898-017-0131-2 (PMC5463304; doi:10.1186/s12898-017-0131-2)
Supplement: Supplementary file 7 — Additional file 7: Table S4. Mean values and standard deviation (StDev) for each trait for each diet of the nutritional geometry for D. biarmipes. [file 12898_2017_131_MOESM7_ESM.docx]

**Table S4** - Mean values and standard deviation (StDev) for each trait for each diet of the nutritional geometry for *D. biarmipes.*

| **Food** | | **Survival** | | **Dev time** | | **Male mass** | | **Female mass** | | **Ovariole** | |
| --- | --- | --- | --- | --- | --- | --- | --- | --- | --- | --- | --- |
| **P:C** | **Cal.** | **Mean** | **StDev** | **Mean** | **StDev** | **Mean** | **StDev** | **Mean** | **StDev** | **Mean** | **StDev** |
| 1:16 | 45 | 0.03 | 0.03 | 354.67 | 78.93 | 0.99 | NaN | 0.71 | NaN | 15.00 | NaN |
| 1:8 | 45 | 0.08 | 0.04 | 296.89 | 59.12 | 0.79 | 0.29 | - | - | - | - |
| 1:4 | 45 | 0.14 | 0.11 | 270.59 | 53.08 | 0.70 | 0.14 | 0.80 | 0.30 | 21.00 | NaN |
| 1:2 | 45 | 0.30 | 0.05 | 214.44 | 44.88 | 0.64 | 0.21 | 0.78 | 0.22 | 20.33 | 3.50 |
| 1:1 | 45 | 0.23 | 0.07 | 183.41 | 27.71 | 0.71 | 0.16 | 0.76 | 0.22 | 17.63 | 2.56 |
| 1.5:1 | 45 | 0.25 | 0.07 | 167.47 | 35.21 | 0.75 | 0.15 | 0.83 | 0.25 | 18.50 | 3.34 |
| 1:16 | 90 | 0.04 | 0.02 | 296.00 | 42.71 | 0.76 | 0.02 | 0.29 | NaN | - | - |
| 1:8 | 90 | 0.09 | 0.07 | 261.09 | 71.49 | 0.77 | 0.16 | 0.83 | 0.13 | 15.00 | 9.90 |
| 1:4 | 90 | 0.22 | 0.10 | 190.15 | 30.55 | 0.88 | 0.11 | 0.97 | 0.18 | 20.25 | 1.71 |
| 1:2 | 90 | 0.47 | 0.16 | 167.86 | 34.84 | 0.73 | 0.14 | 0.88 | 0.12 | 21.85 | 1.77 |
| 1:1 | 90 | 0.73 | 0.15 | 135.54 | 16.83 | 0.83 | 0.11 | 1.03 | 0.16 | 22.92 | 3.00 |
| 1.5:1 | 90 | 0.89 | 0.07 | 126.21 | 13.06 | 0.93 | 0.14 | 1.17 | 0.16 | 23.95 | 2.34 |
| 1:16 | 180 | 0.07 | 0.05 | 272.00 | 62.85 | 0.75 | 0.03 | 0.87 | 0.10 | - | - |
| 1:8 | 180 | 0.09 | 0.03 | 230.55 | 26.49 | 0.89 | 0.06 | 0.96 | 0.08 | 20.00 | 1.41 |
| 1:4 | 180 | 0.47 | 0.11 | 181.29 | 22.54 | 0.81 | 0.09 | 0.95 | 0.11 | 19.00 | 1.85 |
| 1:2 | 180 | 0.87 | 0.05 | 134.69 | 21.01 | 0.95 | 0.10 | 1.16 | 0.15 | 24.62 | 2.04 |
| 1:1 | 180 | 0.93 | 0.06 | 107.86 | 11.41 | 1.08 | 0.12 | 1.30 | 0.10 | 26.23 | 2.12 |
| 1.5:1 | 180 | 0.95 | 0.02 | 105.40 | 11.52 | 1.08 | 0.13 | 1.33 | 0.10 | 26.32 | 1.74 |
| 1:16 | 360 | 0.01 | 0.02 | 392.00 | NaN | - | - | 0.88 | NaN | - | - |
| 1:8 | 360 | 0.13 | 0.12 | 256.00 | 31.42 | 0.62 | 0.08 | 0.85 | 0.13 | 16.00 | 1.41 |
| 1:4 | 360 | 0.87 | 0.05 | 157.67 | 27.66 | 0.96 | 0.10 | 1.11 | 0.16 | 22.29 | 2.20 |
| 1:2 | 360 | 0.88 | 0.03 | 123.55 | 17.56 | 1.05 | 0.15 | 1.32 | 0.10 | 25.93 | 1.95 |
| 1:1 | 360 | 0.86 | 0.06 | 116.19 | 13.71 | 1.10 | 0.06 | 1.38 | 0.13 | 26.20 | 2.30 |
| 1.5:1 | 360 | 0.96 | 0.03 | 114.57 | 15.59 | 1.13 | 0.07 | 1.42 | 0.11 | 26.12 | 1.86 |
