# Supplementary material for: Adaptation to new nutritional environments: larval performance, foraging decisions, and adult oviposition choices in Drosophila suzukii
Source: BMC Ecol. 2017 Jun 7;17:21. doi: 10.1186/s12898-017-0131-2 (PMC5463304; doi:10.1186/s12898-017-0131-2)
Supplement: Supplementary file 8 — Additional file 8: Table S5. Pairwise comparisons between the response surfaces of the five life history traits in Drosophila suzukii. [file 12898_2017_131_MOESM8_ESM.docx]

**Table S5** – Pairwise comparisons between the response surfaces of the five life history traits in *Drosophila suzukii.*

| **Life history trait A** | **Life history trait B** | **D. Freedom** | **L ratio** | ***p*-value** |
| --- | --- | --- | --- | --- |
| Survival | Female mass | 14 | 13.552 | 0.187 |
| Survival | Male mass | 14 | 7.449 | 0.947 |
| Survival | inv. Dev. Time | 14 | 12.362 | 0.241 |
| Survival | Ovarioles | 14 | 5.710 | 1 |
| Female mass | Male mass | 14 | 1.631 | 1 |
| Female mass | inv. Dev. Time | 14 | 12.895 | 0.219 |
| Female mass | Ovarioles | 14 | 6.022 | 1 |
| Male mass | inv. Dev. Time | 14 | 10.191 | 0.210 |
| Male mass | Ovarioles | 14 | 6.034 | 1 |
| Ovarioles | inv. Dev. Time | 14 | 10.802 | 0.388 |

Using partial F tests, we compared the response surfaces generated from linear mixed effects models on the scaled parameter values, using replicates as our random effect. For developmental time, we inverted the data for comparison. The *p* values were adjusted using the Holm’s method.
