# Supplementary material for: Adaptation to new nutritional environments: larval performance, foraging decisions, and adult oviposition choices in Drosophila suzukii
Source: BMC Ecol. 2017 Jun 7;17:21. doi: 10.1186/s12898-017-0131-2 (PMC5463304; doi:10.1186/s12898-017-0131-2)
Supplement: Supplementary file 10 — Additional file 10: Table S7. Mean values (mg) and standard deviation (StDev) for protein and carbohydrate intake for each P:C ratio of the no-choice experiment. [file 12898_2017_131_MOESM10_ESM.docx]

**Table S7** - Mean values (mg) and standard deviation (StDev) for protein and carbohydrate intake for each P:C ratio of the no-choice experiment.

| **Food** | ***D. suzukii*** | | | | ***D. biarmipes*** | | | |
| --- | --- | --- | --- | --- | --- | --- | --- | --- |
|  | **Protein** | | **Carbohydrates** | | **Protein** | | **Carbohydrates** | |
|  | **Mean** | **StDev** | **Mean** | **StDev** | **Mean** | **StDev** | **Mean** | **StDev** |
| 1.5:1 | 0.0104 | 0.0029 | 0.0068 | 0.0019 | 0.0121 | 0.0014 | 0.0080 | 0.0009 |
| 1:1 | 0.0133 | 0.0056 | 0.0133 | 0.0056 | 0.0124 | 0.0027 | 0.0124 | 0.0027 |
| 1:2 | 0.0153 | 0.0020 | 0.0306 | 0.0039 | 0.0249 | 0.0050 | 0.0497 | 0.0099 |
| 1:4 | 0.0185 | 0.0034 | 0.0739 | 0.0135 | 0.0291 | 0.0039 | 0.1164 | 0.0156 |
| 1:8 | 0.0167 | 0.0021 | 0.1335 | 0.0164 | 0.0291 | 0.0028 | 0.2328 | 0.0228 |
| 1:16 | 0.0114 | 0.0027 | 0.1827 | 0.0426 | 0.0208 | 0.0034 | 0.3338 | 0.0540 |
