# Supplementary material for: Adaptation to new nutritional environments: larval performance, foraging decisions, and adult oviposition choices in Drosophila suzukii
Source: BMC Ecol. 2017 Jun 7;17:21. doi: 10.1186/s12898-017-0131-2 (PMC5463304; doi:10.1186/s12898-017-0131-2)
Supplement: Supplementary file 11 — Additional file 11: Table S8. Effects of pair of diets presented, time, larval species and possible interactions for the amount of larvae that chose: Protein rich food; Carbohydrate rich food; Both foods; and None. We analyzed our data with generalized linear models using a quasi-possion distribution (ANOVA type II). [file 12898_2017_131_MOESM11_ESM.docx]

**Table S8** – Effects of pair of diets presented, time, larval species and possible interactions for the amount of larvae that chose: Protein rich food; Carbohydrate rich food; Both foods; and None. We analyzed our data with generalized linear models using a quasi-poisson distribution (ANOVA type II).

|  | **Df** | **Pr(>Chi^2^)** |
| --- | --- | --- |
| **Protein rich Food** |  |  |
| Choice | 2 | **<0.001** |
| Species | 1 | **<0.001** |
| Time | 1 | **<0.001** |
| Choice*Species | 2 | 0.689 |
| Choice*Time | 2 | 0.496 |
| Species*Time | 1 | 0.566 |
| Choice*Species*Time | 2 | 0.381 |
| **Carb. rich Food** |  |  |
| Choice | 2 | **<0.001** |
| Species | 1 | **<0.001** |
| Time | 1 | 0.309 |
| Choice*Species | 2 | **0.005** |
| Choice*Time | 2 | 0.605 |
| Species*Time | 1 | 0.383 |
| Choice*Species*Time | 2 | 0.728 |
| **Both** |  |  |
| Choice | 2 | 0.307 |
| Species | 1 | **<0.001** |
| Time | 1 | **<0.001** |
| Choice*Species | 2 | 0.183 |
| Choice*Time | 2 | 0.211 |
| Species*Time | 1 | 0.419 |
| Choice*Species*Time | 2 | 0.563 |
| **None** |  |  |
| Choice | 2 | 0.590 |
| Species | 1 | **<0.001** |
| Time | 1 | 0.054 |
| Choice*Species | 2 | 0.07 |
| Choice*Time | 2 | 0.204 |
| Species*Time | 1 | 0.972 |
| Choice*Species*Time | 2 | 0.295 |
