# Supplementary material for: Adaptation to new nutritional environments: larval performance, foraging decisions, and adult oviposition choices in Drosophila suzukii
Source: BMC Ecol. 2017 Jun 7;17:21. doi: 10.1186/s12898-017-0131-2 (PMC5463304; doi:10.1186/s12898-017-0131-2)
Supplement: Supplementary file 12 — Additional file 12: Table S9. Effects of pair of diets presented, time, larval species and possible interactions in the amount of protein rich food that third instar larvae chose to consume. We analyzed our data with a generalized linear model using a quasi-poisson distribution (ANOVA type II). [file 12898_2017_131_MOESM12_ESM.docx]

**Table S9** – Effects of pair of diets presented, time, larval species and possible interactions in the amount of protein rich food that third instar larvae chose to consume. We analyzed our data with a generalized linear model using a quasi-poisson distribution (ANOVA type II).

|  | **Df** | **Pr(Chi)** |  |
| --- | --- | --- | --- |
| Choice pair | 2 | **< 0.001** | *** |
| Time | 1 | 0.194 |  |
| Species | 1 | **< 0.001** | *** |
| Choice*Time | 2 | 0.060 |  |
| Choice*Species | 2 | 0.228 |  |
| Time*Species | 1 | **0.030** | * |
| Choice*Time:Species | 2 | 0.852 |  |

Level of significance: *p* < 0.05 * ; *p* < 0.01 ** ; *p* < 0.01 ***
