# Supplementary material for: Adaptation to new nutritional environments: larval performance, foraging decisions, and adult oviposition choices in Drosophila suzukii
Source: BMC Ecol. 2017 Jun 7;17:21. doi: 10.1186/s12898-017-0131-2 (PMC5463304; doi:10.1186/s12898-017-0131-2)
Supplement: Supplementary file 13 — Additional file 13. Table S10. Differences of consumption of each diet for each treatment in D. suzukii and D. biarmipes. We used Wilcoxon signed ranked test each to compare the percentage of protein-rich diet present in the gut to 50% (no-choice). Treatments that show significant differences are highlighted in bold. [file 12898_2017_131_MOESM13_ESM.docx]

**Table S10** – Differences of consumption of each diet for each treatment in *D. suzukii* and *D. biarmipes*. We used Wilcoxon signed ranked test each to compare the percentage of protein-rich diet present in the gut to 50% (no-choice). Treatments that show significant differences are highlighted in bold.

| **Choice pair** | **V statistic** | ***p*-value** | **Choice pair** | **V statistic** | | ***p*-value** |
| --- | --- | --- | --- | --- | --- | --- |
| **2 hours - time point** | |  | **2 hours - time point** | | | |
| ***D. biarmipes*** | | | ***D. suzukii*** | | | |
| 1.5:1 Vs 1:8 | 11 | 0.106 | 1.5:1 Vs 1:8 | 14 | 0.193 | |
| 1:1 Vs 1:8 | 23 | 0.695 | 1:1 Vs 1:8 | 23 | 0.695 | |
| 1:1 Vs 1:16 | 36 | 0.432 | 1:1 Vs 1:16 | 16 | 0.275 | |
| **4 hours - time point** | |  | **4 hours - time point** | | | |
| 1.5:1 Vs 1:8 | 11 | 0.106 | **1.5:1 Vs 1:8** | **0** | **0.002 **** | |
| **1:1 Vs 1:8** | **55** | **0.006 **** | 1:1 Vs 1:8 | 33 | 0.625 | |
| **1:1 Vs 1:16** | **52** | **0.014 *** | 1:1 Vs 1:16 | 10 | 0.084 | |

Level of significance: *p* < 0.05 * ; *p* < 0.01 ** ; *p* < 0.01 ***
