# Supplementary material for: Adaptation to new nutritional environments: larval performance, foraging decisions, and adult oviposition choices in Drosophila suzukii
Source: BMC Ecol. 2017 Jun 7;17:21. doi: 10.1186/s12898-017-0131-2 (PMC5463304; doi:10.1186/s12898-017-0131-2)
Supplement: Supplementary file 14 — Additional file 14: Table S11. Least squared means (Lsmean), standard errors (St. error), and groups for D. suzukii and D. biarmipes nutritional oviposition assays, with significant differences denoted by different numbers in the group column (adjusting p-values using the Bonferroni method for a significance level of 0.05). [file 12898_2017_131_MOESM14_ESM.docx]

**Table S11** - Least squared means (Lsmean), standard errors (St. error), and groups for *D. suzukii* and *D. biarmipes* nutritional oviposition assays, with significant differences denoted by different numbers in the group column (adjusting p-values using the Bonferroni method for a significance level of 0.05).

| **Food Patch** | **Lsmean** | **St. error** | **Group** | **Food Patch** | **Lsmean** | **St. error** | **Group** |
| --- | --- | --- | --- | --- | --- | --- | --- |
| ***D. biarmipes*** | | | | ***D. suzukii*** | | | |
| 1:1 | -2.202 | 0.309 | 1 | 1:1 | -2.385 | 0.186 | 1 |
| 1:4 | -0.841 | 0.202 | 2 | 1:4 | -0.865 | 0.113 | 2 |
| 1:8 | 0.401 | 0.189 | 3 | 1:8 | 0.481 | 0.106 | 3 |
